# Comparing the similarity and difference of three influenza surveillance systems in China

**DOI:** 10.1038/s41598-018-21059-9

**Published:** 2018-02-12

**Authors:** Xiaoting Yang, Dongpeng Liu, Kongfu Wei, Xinfeng Liu, Lei Meng, Deshan Yu, Hongyu Li, Baodi Li, Jian He, Wenbiao Hu

**Affiliations:** 1 Division of Infectious Disease, Gansu Provincial Centre for Disease Control and Prevention, Lanzhou, China; 2 School of Public Health and Social Work, Institute of Health and Biomedical Innovation, Queensland University of Technology, Brisbane, Australia

## Abstract

Three main surveillance systems (laboratory-confirmed, influenza-like illness (ILI) and nationwide Notifiable Infectious Diseases Reporting Information System (NIDRIS)) have been used for influenza surveillance in China. However, it is unclear which surveillance system is more reliable in developing influenza early warning system based on surveillance data. This study aims to evaluate the similarity and difference of the three surveillance systems and provide practical knowledge for improving the effectiveness of influenza surveillance. Weekly influenza data for the three systems were obtained from March 2010 to February 2015. Spearman correlation and time series seasonal decomposition were used to assess the relationship between the three surveillance systems and to explore seasonal patterns and characteristics of influenza epidemics in Gansu, China. Our results showed influenza epidemics appeared a single-peak around January in all three surveillance systems. Time series seasonal decomposition analysis demonstrated a similar seasonal pattern in the three systems, while long-term trends were observed to be different. Our research suggested that a combination of the NIDRIS together with ILI and laboratory-confirmed surveillance is an informative, comprehensive way to monitor influenza transmission in Gansu, China. These results will provide a useful information for developing influenza early warning systems based on influenza surveillance data.

## Introduction

Influenza is associated with considerable mortality and morbidity worldwide. Influenza surveillance systems are important tools for monitoring and evaluating transmission of influenza^1–5^. Gansu is relatively poor province, which public health capacity is backward compared with the economically developed provinces. The influenza incidence rates in Gansu were similar to the national average level. Influenza epidemics in Gansu exhibit strong seasonal winter peaks. In 2009, an extended influenza surveillance system for influenza-like illness (ILI) and virologic data was established in Gansu Province^5^. The extended influenza system aimed to better understand the epidemiologic trend of influenza epidemic and virus variation, and eventually develop an influenza early warning system that could provide advantage for improving the timeliness of epidemic control and formulating scientific prevention strategy. Currently there are three main systems used for influenza surveillance in Gansu Province, China: laboratory-confirmed (i.e., detection of influenza virus nucleic acid), ILI and nationwide Notifiable Infectious Diseases Reporting Information System (NIDRIS). This study aims to evaluate and compare effectiveness of the three influenza surveillance systems and provide practical knowledge for improving the performance of influenza surveillance in Gansu, China.

## Results

### Patterns of seasonality

**Figure 1:**
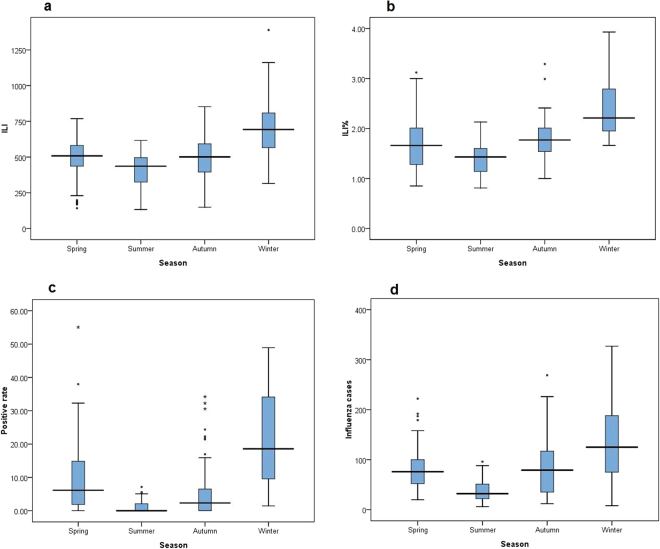
Influenza activity in different seasons in Gansu, 2010–2015. (**a**) Influenza-like illness (ILI) from Chinese Influenza Surveillance Information System. (**b**) ILI consultation rate (ILI%) from Chinese Influenza Surveillance Information System. (**c**) Influenza virus positive rate from Chinese Influenza Surveillance Information System. (**d**) Weekly influenza reported cases from nationwide Notifiable Infectious Diseases Reporting Information System (NIDRIS).

There was a distinct seasonality in influenza occurrence in Gansu Province (Tables 1–3, Fig. 1). Both four series of monitoring data were highest in winter, followed by spring.

**Table 1:** Influenza-like illness from sentinel hospitals in Gansu, 2010–2015.

The annual	ILI	The total number of outpatient and emergency cases	ILI%	Seasons							
				Spring (week 10–22)		Summer (week 23–35)		Autumn (week 36–48)		Winter (week 49–9)	
				ILI	ILI%	ILI	ILI%	ILI	ILI%	ILI	ILI%
2010 Week 10–2011 Week 9	16747	893655	1.87	2618	1.18	2012	0.94	4226	1.94	7891	3.29
2011 Week 10–2012 Week 9	23487	1181779	1.99	6808	2.35	4943	1.75	5286	1.87	6450	1.97
2012 Week 10–2013 Week 9	30602	1533690	2.00	7109	1.86	6096	1.61	7088	1.98	10309	2.49
2013 Week 10–2014 Week 9	30060	1787591	1.68	7690	1.76	6011	1.36	6522	1.51	9837	2.06
2014 Week 10–2015 Week 9	34368	2115479	1.62	7258	1.39	6623	1.24	8834	1.73	11653	2.12
Total	135264	7512194	1.80	31483	1.70	25685	1.39	31956	1.78	46140	2.30

**Table 2:** Laboratory-confirmed influenza from sentinel hospitals in Gansu, 2010–2015.

The annual	The number of specimens	The number of laboratory-confirmed influenza	Positive rate (%)	Seasons			
				Spring (week 10–22)	Summer (week 23–35)	Autumn (week 36–48)	Winter (week 49–9)
2010 Week 10–2011 Week 9	7013	1049	14.96	10.67	0.89	21.89	19.37
2011 Week 10–2012 Week 9	6402	652	10.18	9.10	1.98	3.07	19.66
2012 Week 10–2013 Week 9	6505	1200	18.45	24.87	2.28	7.35	25.16
2013 Week 10–2014 Week 9	9993	1326	13.27	2.03	0.45	1.57	28.99
2014 Week 10–2015 Week 9	12270	1528	12.45	12.12	0.68	2.22	22.92
Total	42183	5755	13.64	11.38	1.09	5.74	23.83

**Table 3:** Influenza cases of nationwide Notifiable Infectious Diseases Reporting Information System (NIDRIS) in Gansu, 2010–2015.

The annual	influenza cases of nationwide Notifiable Infectious Diseases Reporting Information System (NIDRIS)	Seasons			
		Spring (week 10–22)	Summer (week 23–35)	Autumn (week 36–48)	Winter (week 49–9)
2010 Week 10–2011 Week 9	2469	579	216	815	859
2011 Week 10–2012 Week 9	3267	802	311	797	1357
2012 Week 10–2013 Week 9	5399	1407	572	1376	2044
2013 Week 10–2014 Week 9	5519	1163	595	1167	2594
2014 Week 10–2015 Week 9	5248	1481	685	1201	1881
Total	21902	5432	2379	5356	8735

**Figure 2:**
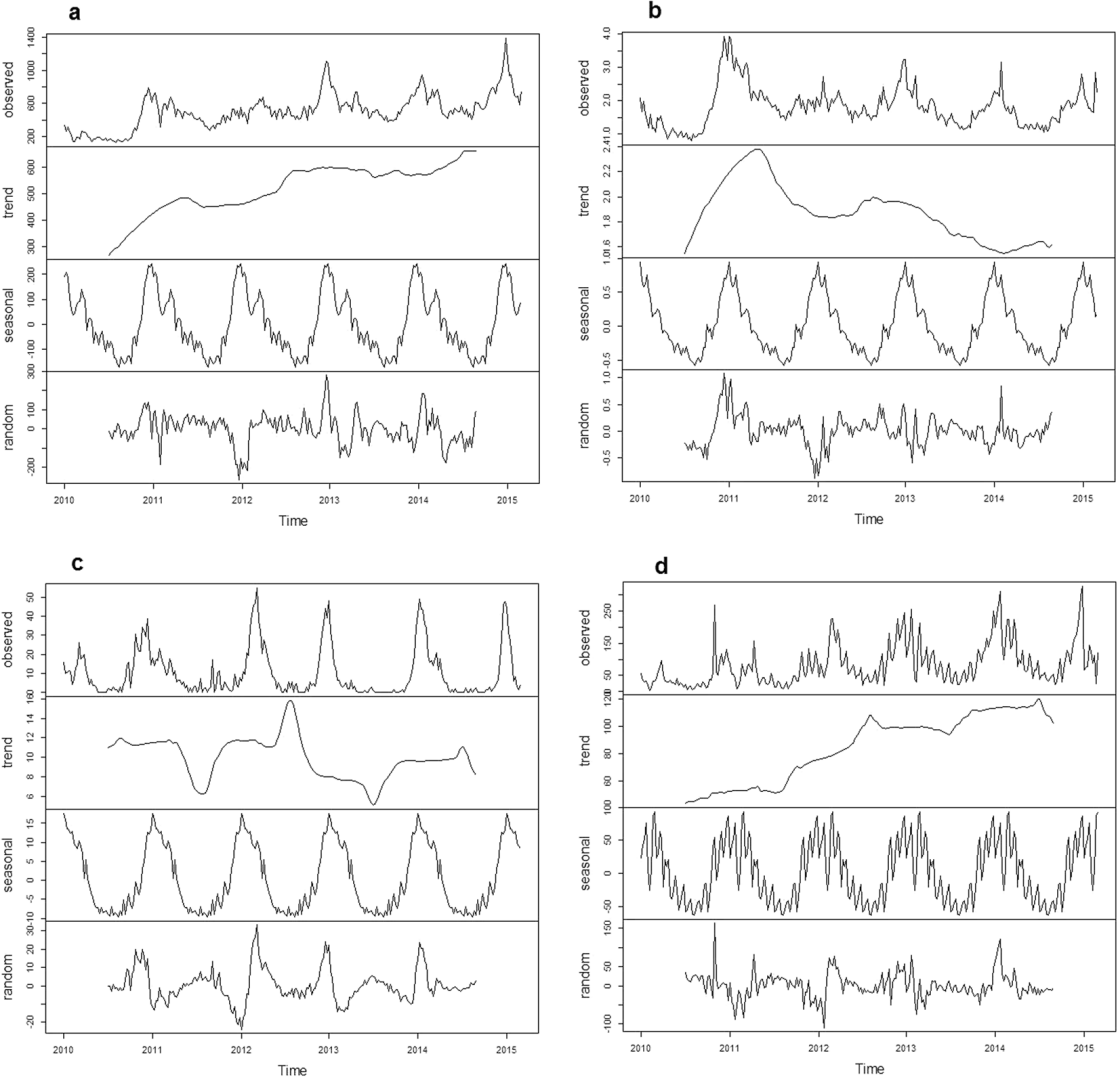
Seasonal decomposition of influenza in Gansu, 2010–2015. (**a**) Influenza-like illness (ILI) from Chinese Influenza Surveillance Information System. (**b**) ILI consultation rate (ILI%) from Chinese Influenza Surveillance Information System. (**c**) Influenza virus positive rate from Chinese Influenza Surveillance Information System. (**d**) Weekly influenza reported cases from nationwide Notifiable Infectious Diseases Reporting Information System (NIDRIS).

Our results showed an influenza epidemic to appear as a typical single-peak, around January, in all three surveillance systems. Time series seasonal decomposition analysis demonstrated a similar seasonal pattern in the three systems (Fig. 2). However, long-term trends were observed to differ between the three systems.

### Correlation analysis

**Figure 3:**
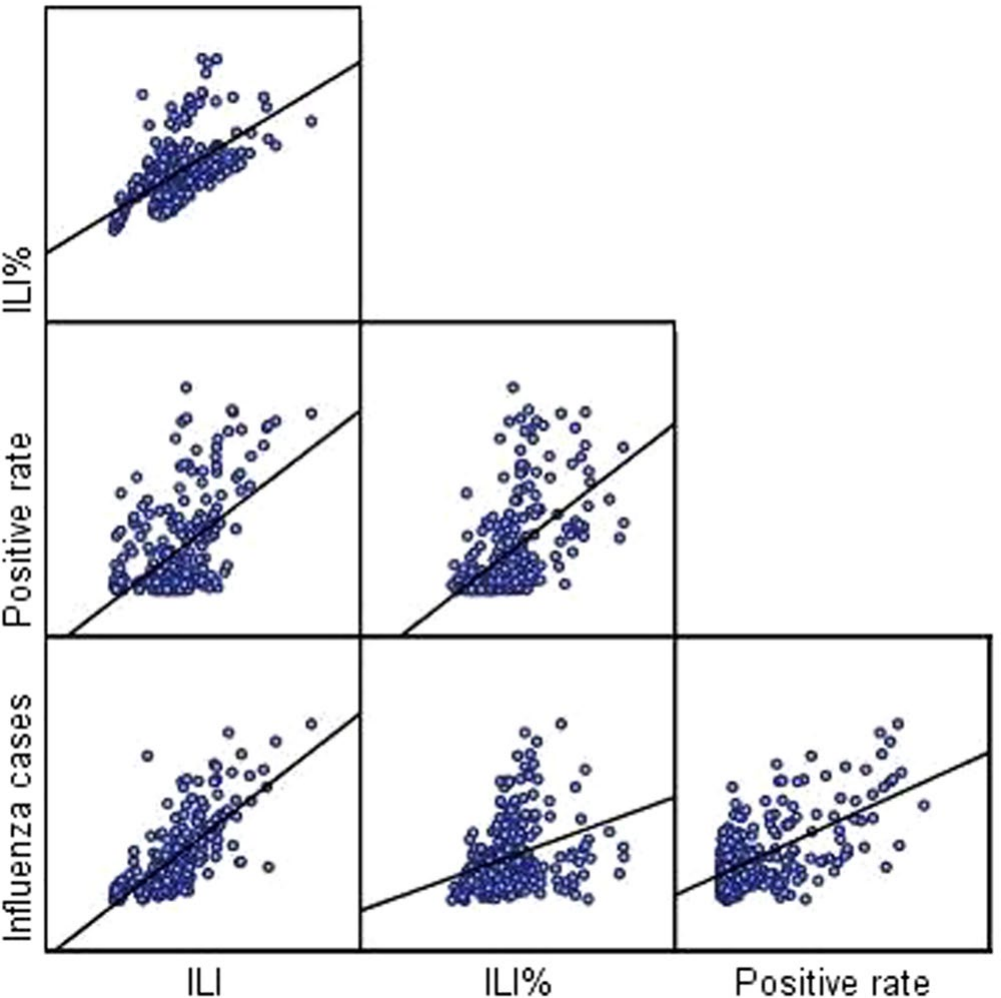
Correlation between different surveillance data in Gansu, 2010–2015. ILI: Influenza-like illness from Chinese Influenza Surveillance Information System. ILI%: ILI consultation rate from Chinese Influenza Surveillance Information System. Positive rate: Influenza virus positive rate from Chinese Influenza Surveillance Information System. Influenza cases: Weekly influenza reported cases from nationwide Notifiable Infectious Diseases Reporting Information System (NIDRIS).

Fitted lines with scatter plots were shown for positive correlation between any two sets of data (Fig. 3). Spearman correlation indicated that influenza cases of NIDRIS correlated significantly with ILI (r = 0.741, p < 0.01), ILI% (r = 0.442, p < 0.01), and positive rate (r = 0.486, p < 0.01) (Table 4).

**Table 4:** Spearman correlation coefficients between different surveillance data in Gansu, 2010–2015.

	ILI	ILI%	Positive rate
ILI%	0.645**		
Positive rate	0.423**	0.596**	
Influenza cases of NIDRIS	0.741**	0.442**	0.486**

**Correlation is significant at the 0.01 level (2-tailed).

### Comparison of advantages and disadvantages

Table 5 summarized the advantages and disadvantages for the three surveillance systems (Table 5).

**Table 5:** Advantages and disadvantages of different surveillance systems in Gansu.

	Data sources	Advantages	Disadvantages	
ILI	Sentinel hospitals	ILI is a better indicator in developing early warning system because it be reported more timely.	ILI-like symptoms may be caused by etiologies other than influenza. Sentinel hospitals report the number of ILI by age group, without case information. Increase in ILI and ILI% might be a result of increased healthcare-seeking behavior after media reports of influenza outbreak or the circulation of non-influenza respiratory viruses.	19 sentinel hospitals are located in urban areas, so the surveillance system may not detect influenza virus activity in rural areas.
ILI%	Sentinel hospitals	ILI% can eliminate the impact of increased number of sentinel hospital.	Long-term trend or short-term changes of the total number of outpatients may impact ILI%, such as “long-holiday effect”.	
Positive rate	Sentinel hospitals	Laboratory surveillance can provide more accurate information about influenza virus activity.	Laboratory test sometimes consumes too much time and thus may delay the early warning of influenza epidemics.	
Influenza cases of NIDRIS	All hospitals	Influenza cases are reported by all medical institutions in Gansu Province, include secondary, tertiary, pediatric hospitals in urban area, and primary hospitals, village clinics in rural area.	Some influenza patients may be miss-diagnosed, because it’s hard to differentiate influenza from other respiratory viruses infection without laboratory testing, especially in the non-epidemic season. As passive surveillance, influenza cases may also be missing-reported.	

## Discussion

There is a high correlation among the three influenza surveillance systems data, suggesting that all three influenza surveillance systems in Gansu Province reflect the epidemic characteristics of influenza. The three influenza surveillance systems indicated that there were epidemics peaks in winter and similar seasonal pattern. The influenza epidemics typically appeared as a single-peak around January in Gansu; this finding is consistent with other northern provinces in China^1,6^. Seasonal decomposition, however, showed differences in long-term trends of the three systems. The increase in ILI cases may be due to surveillance sensitivity improvement, while the downward trend of Influenza-like illness consultation rate (ILI%) may be due to the increase in total visits to outpatient and/or emergency departments. At the same time, the increase in monitoring sensitivity, and the increase in laboratory-confirmed influenza cases resulted in an increase in reported cases of influenza from NIDRIS. Influenza virus positive rate, the percentage of swabs that are positive for influenza by real-time Polymerase Chain Reaction (PCR), can provide accurate information about influenza virus activity, while its monitoring results indicated that influenza epidemic peak didn’t occur in the very same time (Fig. 2c), so it’s very necessary to carry out continuous monitoring of influenza and set up a suitable early warning technology^7–10^.

Each of the three surveillance systems has its own advantages and disadvantages. Laboratory surveillance can provide more accurate information about the activity of influenza virus serotypes, so influenza virus positive rate could be used as an indicator for influenza epidemic; it is, however, time consuming and resource intensive and thus may delay the early warning and control of influenza epidemics^11^. By contrast, ILI and ILI% are better indicators for use in early warning systems as they tend to be more time efficient in the generation of data. Sentinel systems, however, rely upon individual doctors or clinics to collate data and report it to the relevant authority, this may introduce compliance or bias issues. Furthermore, illnesses other than influenza may present with ILI^12^; sentinel hospitals report ILI by age group, without case information; increases in ILI and ILI% may be the result of increased healthcare-seeking behavior in response to media reports or the circulation of non-influenza respiratory viruses^13,14^. ILI, ILI% and positive rate are based on the sentinel surveillance network which is built on data sourced from urban areas; as such, this system may not detect influenza virus activity in rural areas^2,4,11,15,16^. By contrast, influenza cases of NIDRIS are reported by all medical institutions in Gansu Province, including secondary, tertiary, pediatric hospitals in urban areas, and primary hospitals and village clinics in rural areas. Weekly influenza data from the NIDRIS were observed to have larger fluctuations and during the long vacation (especially Chinese New Year), influenza cases greatly reduced (Fig. 2d) and data stability were affected because of an obvious decrease of patients’ visits. Some influenza patients may be misdiagnosed, because it’s hard to differentiate influenza from other respiratory viruses without laboratory testing, especially in the non-epidemic season; in addition, as passive surveillance, influenza cases may also be misreported to NIDRIS. This demonstrated the need to improve mechanisms for sharing information between influenza surveillance systems^4,17^, to enhance capacities.

This study was the first attempt to compare performance of all the three influenza surveillance systems in Gansu Province. The results of this study provide practical knowledge for developing early warning systems for influenza based on surveillance system with climate and socio-environmental data^18^.

As the influenza surveillance systems were extended in 2009, only five-year data time series were available. This limited analysis of long-term epidemiological characteristics^19,20^.

## Conclusion

A combination of the NIDRIS together with ILI and laboratory-confirmed surveillance is an informative, comprehensive way to monitor influenza transmission in Gansu, China. We should consider combining the three surveillance systems in developing influenza early warning systems.

## Methods

Located in the northwest of China, Gansu Province covers an area of 453,700 square kilometers, with a population of 25.91 million (2014). The climate is cold and dry in Gansu Province and exhibits large fluctuations in temperature between day and night. The annual average temperature ranges from 0 degrees Celsius in the northwest to 14 degrees Celsius in the southeast; mean annual rainfall is about 300 millimeters, however this differs greatly across regions.

Weekly ILI data were obtained from the national sentinel hospital-based influenza surveillance network. This network consists of 19 sentinel hospitals in Gansu, dispersed through 18 counties and districts, including all 14 cities of Gansu. These 19 sentinel hospitals are the larger comprehensive hospitals, located in the densely populated districts of these cities, and are recognized by the National Ministry of Health to represent the medical-seeking people in Gansu. Each week, participating doctors in the outpatient and emergency departments of internal medicine and pediatrics were required to report the number of patients with ILI by age group (i.e., 0–4 years, 5–14 years, 15–24 years, 25–59 years, and >60 years), and total visits to outpatient and/or emergency departments to the Chinese Influenza Surveillance Information System, which is a centralized online system. Identification of patients with ILI was based on a standard case definition, including body temperature ≥38 °C with either cough or sore throat, in the absence of other laboratory-confirmed evidence^21^. In this study, surveillance data for the period of March 2010 (2010 week 10) to February 2015 (2015 Week 9) was used. ILI consultation rate (ILI%) = ILI/total visits to outpatient and emergency departments * 100%.

Laboratory-confirmed influenza cases were collected for same period from the Chinese Influenza Surveillance Information System. In each sentinel hospital, nasopharyngeal swabs were collected from patients who presented within 3 days of ILI onset. The specimens were transported in viral transport media at 4 °C to the diagnostic laboratories within 48 h of collection. Analysis for influenza virus nucleic acid was performed by real-time Polymerase Chain Reaction (PCR) within 1 week. Taking swabs is a mandatory practice for all sentinel hospitals. Each hospital was required to provide 5 to 15 specimens per month from April to September and 10 to 15 specimens per week from October to next March^21^. Participating doctors chose the patients with typical ILI symptom to collect their swabs, and ensure that the specimen was evenly distributed every week or month.

Influenza is a notifiable infectious disease in China^22^. Case definitions for influenza and diagnostic criteria are outlined by the National Health and Family Planning Commission of the People’s Republic of China, which includes clinically diagnosed cases and laboratory-confirmed cases. Notifications of cases are made to NIDRIS, by all medical institutions in Gansu Province, including secondary, tertiary and pediatric hospitals in urban areas, primary hospitals and village clinics in rural areas. Weekly data for reported cases of influenza were obtained from NIDRIS for the period of March 2010 (2010 week 10) to February 2015 (2015 Week 9).

The three systems were used to validate the influenza surveillance quality each other, especially in epidemic period.

Four sets of monitoring data were obtained from the three influenza surveillance systems: ILI, ILI%, positive rate of nucleic acid detection and influenza cases of NIDRIS. The sets of ILI and ILI% were taken from influenza-like illness surveillance of Chinese Influenza Surveillance Information Systems, the set of positive rate came from the laboratory testing results of Chinese Influenza Surveillance Information Systems, while the set of influenza cases was obtained from reported influenza numbers of NIDRIS, including all the clinically diagnosed cases and laboratory-confirmed cases. All four series data were collected continuously from March 2010 (2010 week 10) to February 2015 (2015 week 9). Each series of data was up to five years.

Spearman correlation was used to assess the relationship between the three surveillance systems, as the data appeared to have non-normally distributed pattern. A seasonal decomposition analysis was conducted to assess whether there was a distinct seasonality in each series. In this analysis, each time series was decomposed into seasonality, long-term trend, and irregular factors. The long-term trend showed progression of the influenza series (secular variation). A trend exists when there was a persistent increasing or decreasing direction in the surveillance data^23^.

We confirm that all methods were performed in accordance with the relevant guidelines and regulations by including a statement in the methods section.

### Ethics approval and consent to participate

The study was approved by the Research Ethics Committee of Gansu Provincial Center for Disease Control and Prevention. Informed consent is not relevant to the study as this study used aggregated data without personal information.

### Availability of data and material

Please contact author for data requests.
